# Acoustic Indoor Localization Augmentation by Self-Calibration and Machine Learning

**DOI:** 10.3390/s20041177

**Published:** 2020-02-20

**Authors:** Joan Bordoy, Dominik Jan Schott, Jizhou Xie, Amir Bannoura, Philip Klein, Ludwig Striet, Fabian Hoeflinger, Ivo Haering, Leonhard Reindl, Christian Schindelhauer

**Affiliations:** 1 Department of Computer Science (IIF), University of Freiburg, 79110 Freiburg, Germany; jizhou.xie@outlook.com (J.X.); philipkln.91@gmail.com (P.K.); ludwig.striet@googlemail.com (L.S.); schindel@informatik.uni-freiburg.de (C.S.); 2 Department of Microsystems Engineering (IMTEK), University of Freiburg, 79110 Freiburg, Germany; dominik.jan.schott@imtek.uni-freiburg.de (D.J.S.); reindl@imtek.uni-freiburg.de (L.R.); 3 Department of Software Engineering, Bethlehem University, P.O. Box 11407, Bethlehem, 92248 Jerusalem, Palestine; abannoura@bethlehem.edu; 4 Fraunhofer Institute for Highspeed Dynamics, Ernst-Mach-Institute (EMI), 79104 Freiburg, Germany; Fabian.Hoeflinger@emi.fraunhofer.de (F.H.); ivo.haering@emi.fraunhofer.de (I.H.)

**Keywords:** self-calibration, localization, ultrasound, machine learning, indoor localization, tdoa, random forest

## Abstract

An acoustic transmitter can be located by having multiple static microphones. These microphones are synchronized and measure the time differences of arrival (TDoA). Usually, the positions of the microphones are assumed to be known in advance. However, in practice, this means they have to be manually measured, which is a cumbersome job and is prone to errors. In this paper, we present two novel approaches which do not require manual measurement of the receiver positions. The first method uses an inertial measurement unit (IMU), in addition to the acoustic transmitter, to estimate the positions of the receivers. By using an IMU as an additional source of information, the non-convex optimizers are less likely to fall into local minima. Consequently, the success rate is increased and measurements with large errors have less influence on the final estimation. The second method we present in this paper consists of using machine learning to learn the TDoA signatures of certain regions of the localization area. By doing this, the target can be located without knowing where the microphones are and whether the received signals are in line-of-sight or not. We use an artificial neural network and random forest classification for this purpose.

## 1. Introduction

The applications for which indoor localization systems are envisioned are immense. Among others, these systems can be used in intralogistics. The domain of in-house logistics of commercial enterprises, sometimes also referred to as intralogistics, covers the entire range of organization, execution and optimization of in-house material flow and warehousing. While traditionally, and by the means of material transportation, a static infrastructure is understood, involving conveyor belts, manually operated cranes and fork lifts, there is a trend towards autonomous agents. An important factor is localization and tracking of these autonomous systems [1,2].

Today, many different indoor localization systems are available based on different methods. A large number of them use radio frequency (RF) signals for localization [3,4,5,6]. The RF-Systems use the propagation of radio waves. Another possibility is to use the propagation of sound for localization [7,8]. Indoor localization systems work with indoor satellites (anchor nodes), which have to be installed inside the indoor environment. Most of the traditional indoor localization methods assume that the position of these anchor nodes are known in advance. By knowing their position, the position of the target can be estimated. However, this means that in a real-life scenario, they have to be manually measured, which is a cumbersome job and prone to errors. Therefore, a system which is capable of locating the target without manually measuring the anchor node positions is necessary. We propose two different methods. The first method estimates the positions of the anchor nodes and the target using non-linear optimization. This allows locating the target with an error in the order of centimeters. This method increases the success rate of traditional self-calibration methods by using an inertial measurement unit (IMU) as an additional source of information. Moreover, it is robust against non-line-of-sight measurements, as it discretizes the solution space, choosing the discretized solutions which comply with the information gathered by the IMU.

We propose another method which uses machine learning to identify when the target is in predefined regions. This does not allow localization outside these regions. However, it ensures proper localization in the zones of interest. This is because one can locate the target independently of whether the received signals are in line-of-sight or non-line-of-sight. The algorithm learns from the measurements that are received in the region regardless of this matter. Moreover, the installation effort is reduced even more than estimating the anchor node positions. This is because when estimating the anchor node positions, one needs to ensure that all receivers get enough line-of-sight measurements from different positions in the location area, so that their position can be estimated. Moreover, one needs to ensure that the graph of estimated positions is rigid. This means, there needs to be enough sender positions so that all receivers are constrained by the same coordinate system without allowing any rotation or translation.

In this work, we present a novel method for self-calibration, which determines the anchor positions before the localization of the target. The goal is to improve the success rate of the traditional self-calibration methods. The novel algorithm that is presented in this study simplifies the calibration of acoustic localization systems without compromising the robustness of the system. This is due to the fact that the information gathered from the inertial measurement unit is used to refuse unlikely distributions of receivers.

Additionally, in this manuscript, we show how machine learning algorithms can be used for locating a target when the receiver positions are unknown. This is specially useful in situations where one wants to know whether a speaker is in a certain area. By training the algorithm over different regions, one can then identify in which of the predefined regions the target is located. The results suggest that this method can be applied for zone detection in real-life scenarios.

## 2. State of the Art

In classic hyperbolic TDoA localization, the positions of static receivers are assumed to be known in order to locate a moving sender. In the scope of this work, we will refer to the stationary nodes as anchors, and as beacons to the localized nodes. In some publications, it is suggested to compensate for given erroneous reference anchor positions [9,10], but in general, the positions of anchors are assumed to be precisely determined by external means in an external coordinate system. Manual measurement of anchor positions may be done by a measuring tape, a laser range finder, a grid arrangement or specifically structured sensor array, or by geodetic methods.

The problem of calibration-free TDoA is challenging. First, the number of unknown variables to be determined is higher than in conventional TDoA, as the anchor positions need to be estimated. Second, the beacon and anchor positions depend on each other, which is adverse to the robustness of the estimation. For instance, one failed measurement at a specific time, a large outlier, affects the estimation of anchor positions, which again affect the estimation of other sender positions. In contrast, in conventional TDoA, a failed measurement can only influence a single sender position. Third, due to the high dimensionality of the problem, many strategies to linearize the problem [11,12] cannot be applied in general, so iterative approaches are required, which are time-consuming and require careful initialization.

For self-calibration, different methods are available. In [13], Biswas and Thrun apply a maximum likelihood estimation algorithm to localize the anchors and the beacon simultaneously. In [14], Wendeberg et al. present a non-linear optimization approach for TDoA self-calibration. In the self-calibration problem, both beacon locations and anchor positions are unknown variables. Starting from the randomized initial values, Wendeberg et al. use gradient descent and the Gaussian–Newton method to search for a minimum of the TDoA objective function.

Due to the non-convexity of the hyperbolic function, there may be multiple local minima. Thus, the non-linear optimization method does not ensure a global minimum. In [14], Wendeberg et al. repeat the randomized initialization to increase the probability of finding the global minimum. Furthermore, in [15], Wendeberg et al. solve the self-calibration problem with a branch-and-bound algorithm. In comparison to the optimization-based method, the branch-and-bound method requires more computational power, as revealed in [16]. In [17,18], a far-field assumption is used to simplify the equations and initialize the variables. In contrast, here we present an initialization technique which utilizes the gyroscope measurements to generate an initial guess close to the true positions. It improves the success rate of self-calibration and increases the converging rate during the optimization process. For the sake of convenience, we name this technique direction adjudgment, or DA in short, and we use DA-SC to refer to the self-calibration initialized by direction adjudgment. Meanwhile, in this work, RA refers to the randomized initialization and RI-SC refers to the self-calibration initialized by randomized initialization.

In this work, we also explore the possibility of using machine learning for detecting whether a speaker is located in some predefined areas, without having to measure or estimate the positions of the anchors. Some authors have explored the possibility of using these algorithms for TDoA localization in the past. For example, Niitsoo et al. [19] use the channel impulse responses for localization. Feig et al. [20] use recurring neural networks on drifting time-of-flight measurements. Zhang et al. [21] use a support vector machine (SVM) for identifying whether an acoustic measurement is in line-of-sight or non-line-of-sight. Ebrahimkhanlou et al. [22] use deep learning to locate acoustic emission sources in plate-like structures.

## 3. Localization by TDoA with Machine Learning

A simple but effective approach to avoid the tenuous manual measurement of all $N_{k}$ anchors’ three-dimensional positions is to use a machine learning algorithm in order to learn the characteristic TDoAs at each position. This is specially useful in situations where the positional error is less important than a correct association of the position information, e.g., if a customer is at a certain table. Therefore, we define two-dimensional rectangular clusters that mimic this situation. In order to train the algorithms, we put the transmitting beacon in multiple positions b inside every cluster and save the first reception time $t_{r}$ of every anchor. We train a neural network and a random forest classifier with TDoA vectors τ that contain all the possible anchor pairing permutations. We train both so that we can compare their performance over different circumstances.

### 3.1. Time Difference of Arrival (TDoA)

Having one of the $N_{k}$ anchors with index *i* located at a three-dimensional position $k_{i}$ and a speaker located at a position b, the distance between the two nodes is described in Equation (1). Consequently, a signal traveling from b to $k_{i}$ is received at time $t_{i}$ as in Equation (2).

(1) $$\begin{array}{c} r_{bi}=∥k_{i}-b∥ \end{array}$$

(2) $$\begin{array}{c} t_{i}=t_{b}+\frac{r_{bi}}{c} \end{array}$$

where *c* is the sound velocity and $t_{b}$ the starting time of the signal transmission. Then, having another one of those anchors with index *j* at position $k_{j}$, one can calculate the TDoA $\tau_{ij}$ as formulated in Equation (3), which does not depend on the sending time.

(3) $$\begin{array}{c} \tau_{ij}=t_{i}-t_{j}=\frac{r_{bi}-r_{bj}}{c},\;i,j\in \left[1,...,N_{k}\right]\;|\;i<j \end{array}$$

In order get a notion of how large the clusters can be, one can study what is the minimum achievable position error using TDoA and assuming every timestamp has additive Gaussian noise with standard deviation $\sigma_{n}$.

When using hyperbolic multilateration, two timestamps are used for every measurement, which means the noises between multiple measurements are correlated. As shown in [23], one can define a matrix D as:

(4) $$D=\left(\begin{array}{ccccc} -1 & 1 & 0 & \cdots & 0\\ -1 & 0 & 1 & \cdots & 0\\ \vdots & \vdots & \ddots & \vdots & \vdots \\ -1 & 0 & 0 & \cdots & 1 \end{array}\right)$$

The first column corresponds to the reference anchor whose timestamp is subtracted to the others. The other $N_{k}-1$ columns correspond to the other anchors. Then the $\left(N_{k}-1\right)\times \left(N_{k}-1\right)$ noise matrix of the TDoA measurements will be:

(5) $$R_{\mathrm{TD}}=\sigma_{n}^{2}DD^{T}$$

Then, the matrix A which contains an estimation of the covariance of the estimated variables can be calculated as follows:

(6) $$A=({H^{T}R_{\mathrm{TD}}^{-1}H)}^{-1}$$

where **H** is the Jacobian matrix of the TDoA sensor model. The squared root of the trace of A is a lower bound for the achievable root mean squared error assuming a non-biased estimator:

(7) $$\mathrm{RMSE}\left(\hat{b}\right)\geq \sqrt{\mathrm{Tr}\left(A\right)}$$

where $\hat{b}$ is the estimation of b. Moreover, if this term is normalized by the noise, it is known as the dilution of precision and is used to determine how the geometrical distribution of anchors affects the final position estimation:

(8) $$\mathrm{DOP}=\sigma_{n}\sqrt{\mathrm{Tr}\left(A\right)}$$

### 3.2. Neural Network Classification

**Figure 1:**
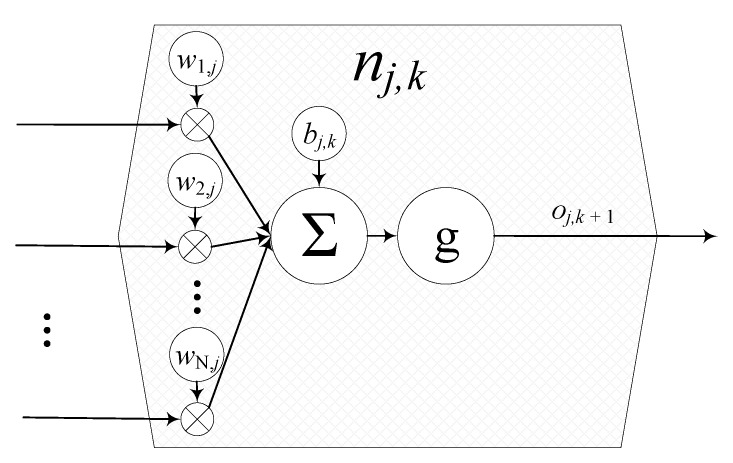
Schematic of a neuron, that can be joined with others to form an artificial neural network.

**Figure 2:**
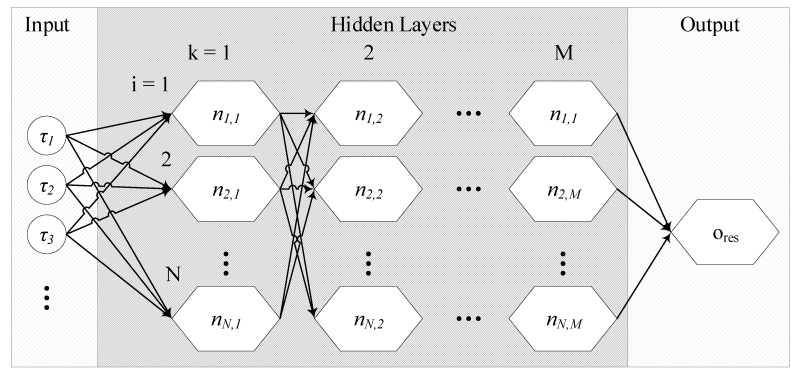
Schematic of an artificial neural network.

An artificial neural network consists of a high number of neurons *n* which are interconnected over a certain number *M* of layers. The layers between the input and the output layer are the hidden layers and determine the complexity of the functions which they approximate (see Figure 1 and Figure 2). Then, the connection between a neuron *i* of the layer k-1 with another neuron *j* of the next layer *k* is weighted with a weight $w_{i,j}$ before being added to the other weighted inputs of the neuron. Afterwards, each neuron adds a bias $b_{j,k}$. The output $o_{j,k}$ of the neuron *j* in the layer *k* is calculated by the activation function g(·). This can be written as follows [19]:

(9) $$\begin{array}{c} o_{j,(k+1)}=g\left(b_{j,k}+\sum_{i=0}^{N}w_{i,j}n_{i,k}\right) \end{array}$$

where *N* is the number of neurons per layer. The value of $n_{i,k}$ is the activation of a neuron in the previous layer.

### 3.3. Random Forest Classification

**Figure 3:**
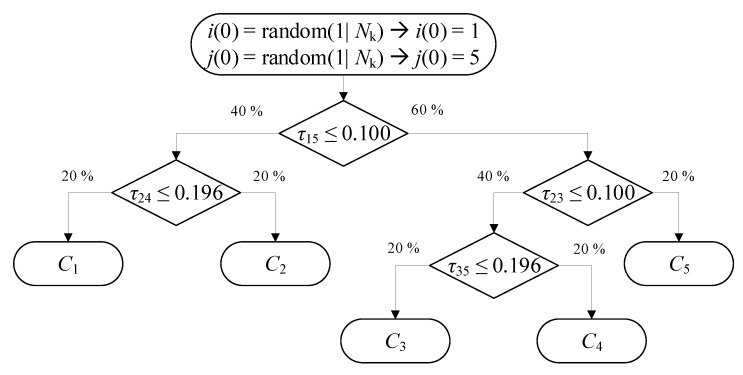
Simple example of a decision tree with 5 clusters. Depending on the TDoA measurements a cluster can be chosen.

The random forest consists of multiple trained trees. These trees have multiple splits where the data gets separated into two similar sized groups. Possible criteria for the split are the gini index or the entropy. See Figure 3 for a simple example with five clusters $C_{1}$ to $C_{5}$. In each branch, we have a simple condition based on one TDoA measurement. If one would use only one tree, a problem would arise. If we do not have any data about the root node or any node along the way, the tree cannot make a prediction, or at least, it can only be used to analyse a subset of clusters. To work around this problem, we do not train one tree but a forest of trees. All trees are trained on different subsets of the training data and use different splits to distinguish between the clusters. As well as reducing our reliance on each TDoA measurement, the ensemble of trees also reduces the bias that a single trained tree has. Another advantage of the random forest is that it is very easy to interpret as we can look into the splitting points and see how the clusters get separated.

## 4. IMU-Assisted Self-Calibration Algorithm

In this case, we aim to estimate the unknown variables: the position of the beacon b and the position of the anchors $k_{i}$. In order to avoid that the non-linear optimization algorithms get stuck in local minima, we first estimate a suitable initial value for these variables by additionally measuring the rotational speeds with a gyroscope; in our case, embedded in an IMU. While the additional sensor generally requires a separate calibration on its own and increases the number of error sources, the proposed method only uses the gyroscope data for a rough estimation that is less influenced by the sensor error. Further signal pre-conditioning of the gyroscope data can be implemented to increase this estimation accuracy and limit the search interval for each anchor even further [24].

The proposed initialization algorithm assumes that at the beginning, the beacon is located under an anchor. Consequently, one can estimate the distance to other three anchors. By knowing these distances, one can define the coordinate system in such a way that two anchors are fixed and the other two must lie each one in a different circle. The solutions inside such circles are discretized into equally spaced points. When the target moves, it generates measurements which must be consistent with the estimated positions. The measurements from the anchors and the ones from the gyroscope are used to decide the most likely solutions inside the discretized solution space. Once the positions of the first four anchors have been estimated, the other anchors can also be located using the estimated sender positions. The details of this algorithm are provided in the next sections.

### 4.1. Anchor Distances and Possible Space Reduction

**Figure 4:**
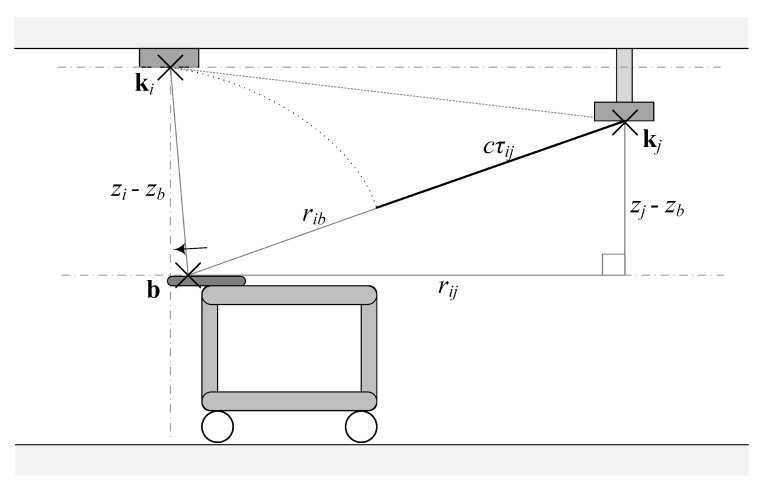
Distance estimation set-up, that reduces the degrees of freedom.

The unknown anchors are assumed to be located on two-dimensional planes with known fixed heights. Hence, the possible space of each anchor is a horizontal plane which has two degrees of freedom. If we take one anchor at position $k_{i}$ as the pivotal reference point and estimate the horizontal distance to another encompassing anchor at position $k_{j}$, we can reduce the possible space of the latter to a circle whose midpoint is $k_{i}$ and its radius $r_{ij}$ equals to the geometric distance between the two positions, as formulated in Equation (10). Note that $r_{ij}$ is the projection of the geometric distance onto the lateral plane in the height of the beacon, in contrast to the distance between beacon and anchors (compare Equation (1)). This is also illustrated in Figure 4.

(10) $$\begin{array}{c} r_{ij}=∥k_{j}-k_{i}∥ \end{array}$$

In order to estimate the distance on the two-dimensional plane between those two anchor positions, we place the sender vertically below the anchor at the reference position. This allows for a simplified calculation of the time difference of arrival between the reference anchor and encompassing anchor.

(11) $$\begin{array}{c} r_{ij}=\sqrt{{\left(r_{bj}\right)}^{2}-{\left(r_{bi}\right)}^{2}} \end{array}$$

(12) $$\begin{array}{cc} r_{bi} & =\sqrt{{\left(x_{i}-x_{b}\right)}^{2}+{\left(y_{i}-y_{b}\right)}^{2}+{\left(z_{i}-z_{b}\right)}^{2}} \end{array}$$

(13) $$\begin{array}{cc} r_{bj} & =r_{bi}+c\;{\bar{\tau }}_{ij} \end{array}$$

**Figure 5:**
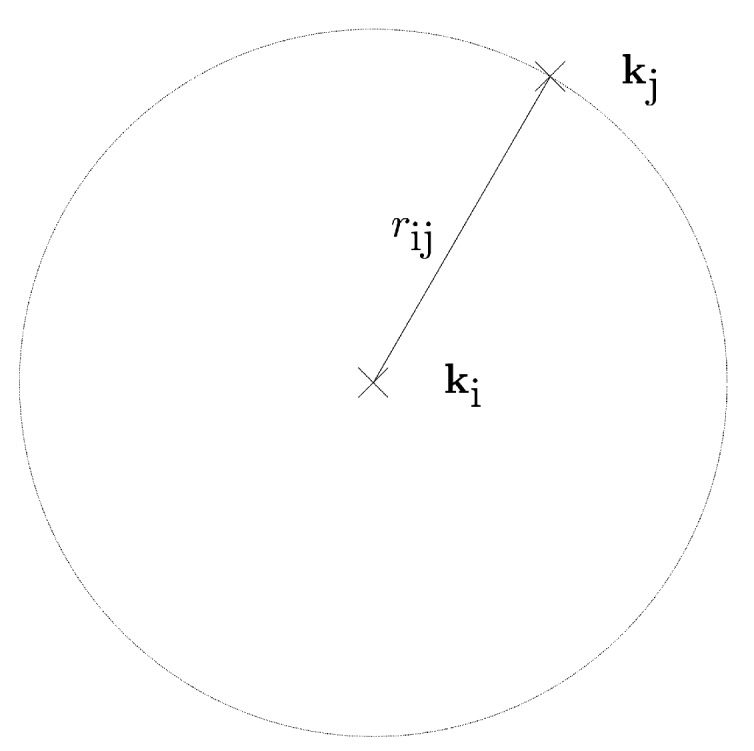
Knowing the distance between the pivotal anchor and the encompass anchor, the pivotal anchor must lie in a circle whose center is the encompass anchor.

Let ${\bar{\tau }}_{ij}$ be the mean value of the TDoA measurements and $z_{i}$, $z_{j}$ and $z_{b}$ be the heights, we can regard the distance $r_{bi}$ in this specific arrangement as a entirely vertical and one-dimensional problem, as shown in Equation (14).

(14) $$\begin{array}{cc} \lim_{x_{b}\to x_{i},\;y_{b}\to y_{i}}r_{bi} & =z_{i}-z_{b} \end{array}$$

(15) $$\begin{array}{cc} \lim_{x_{b}\to x_{i},\;y_{b}\to y_{i}}r_{bj} & =z_{i}-z_{b}+c\;{\bar{\tau }}_{ij} \end{array}$$

and thus we can express the horizontal distance $r_{ij}$ (see Figure 5) as the geometric distance formulated in Equation (16).

(16) $$\begin{array}{c} r_{ij}=\sqrt{{\left(z_{i}-z_{b}+c\;{\bar{\tau }}_{ij}\right)}^{2}-{\left(z_{i}-z_{b}\right)}^{2}}. \end{array}$$

As the anchors are on the ceiling and our vertical coordinate increases from floor to ceiling, we assume that $z_{i}\approx z_{j}\gg z_{b}$ always holds.

Equations (14) and (16) are only valid when the condition that the beacon is right under the pivotal anchor holds. We observed with a Motion Capture system [25] that the placement error due to our visual estimation remains approximately below 0.3 m to the actual position of the anchors. It is important to note that the error of human observation in this phase only influences the initialization of the variables. The aim is to find an initial value for the variables of the scenario which can then be refined with nonlinear optimization without getting stuck in local minima.

As mentioned before, the possible space of the anchor can be reduced to a circle if its distance to the pivotal anchor $r_{1,i}$ is known. We further discretize the circle as discontinuous points on the circle. As the encompass anchor is at the coordinate origin in the *x* and *y* coordinates, the discretized positions of the pivotal anchors $k_{i}^{-}$ are defined in Equation (17).

(17) $$\begin{array}{c} k_{i}^{-}=\left(\begin{array}{c} r_{1,i}\;\cos\;\left(\phi_{i,k}\right)\\ r_{1,i}\;\sin\;\left(\phi_{i,k}\right)\\ z_{i} \end{array}\right), \end{array}$$

where $z_{i}$ is the known height of the anchor, $r_{1i}$ is the estimated inter-anchor distance and $-\pi ⩽\phi_{i,k}<\pi$. We discretize the angle as steps of Δϕ:

(18) $$\begin{array}{c} \phi_{i,k}=k\;\Delta \phi \end{array}$$

where k∈Z. Here Δϕ decides the resolution of the discretized approximation.

**Figure 6:**
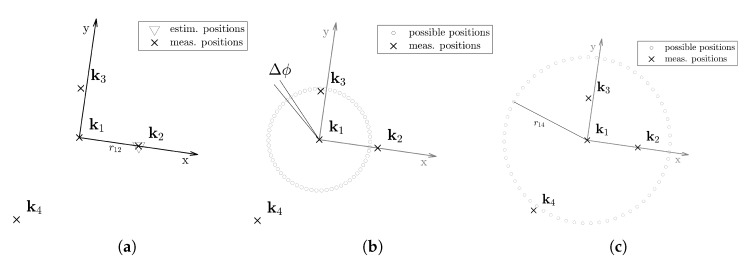
Reduced possible spaces for four anchors. In (**a**) we establish a new coordinate system. The pivotal anchor $k_{1}$ is fixed at the origin in *x* and *y*. The estimation of the anchor $k_{2}$ is fixed at $x=r_{12}$, y=0. The possible space of the additional anchors $k_{3}$ in (**b**) and $k_{4}$ in (**c**) are on circles around the origin.

In order to reduce the computational complexity, we avoid the congruent transformation of anchor configurations. We establish a coordinate system where the pivotal anchor is fixed at the origin and one of the encompass anchors on the positive half of the *x*-axis, i.e., at the point $k_{2}=\left[r_{12},0\right]$, where $r_{12}$ is the estimated distance between the pivotal anchor $k_{1}$ and the first encompassing anchor $k_{2}$. The possible spaces of the other two anchors $k_{3}$ and $k_{4}$ are circles around the pivotal anchor. Figure 6 gives an example for the first four anchors.

### 4.2. Straight Movement Detection

In order to further reduce the subspace of possible solutions $k^{-}$, we use combinations of four anchors from the subspace and estimate the sender positions. Afterwards, we compare the results with the measurements from the inertial measurement unit and discard the anchor positions which lead to sender positions which contradict the IMU estimations. In order to do so, we use the inertial measurement unit to detect when the target is moving in a straight line. The heading direction remains unchanged when this happens. Therefore, the horizontal rotation is close to zero. We first smooth the rotation measurement $\omega_{z}$ and then apply a threshold on the smoothed curve. The endpoints of the straight-moving intervals can be found around the intersections of the threshold line and the smoothed $\omega_{z}$ curve.

**Figure 7:**
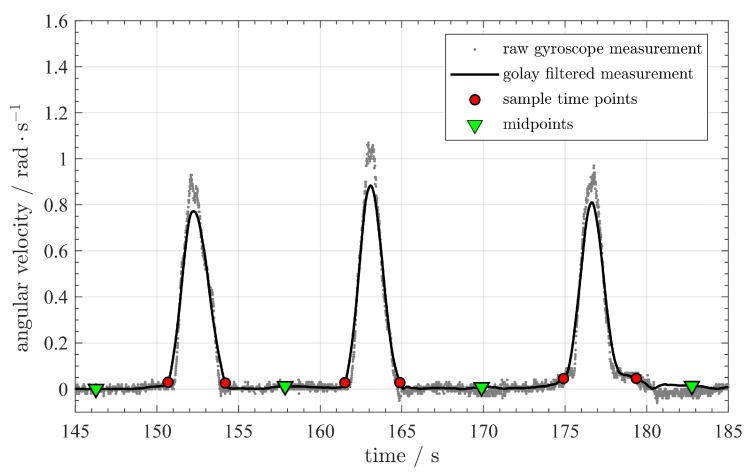
Extracting the time interval when a beacon moves in straight lines. The magenta points denote the sample points where the straight moving interval starts and ends. The green points are the midpoints of time in the straight moving intervals.

**Figure 8:**
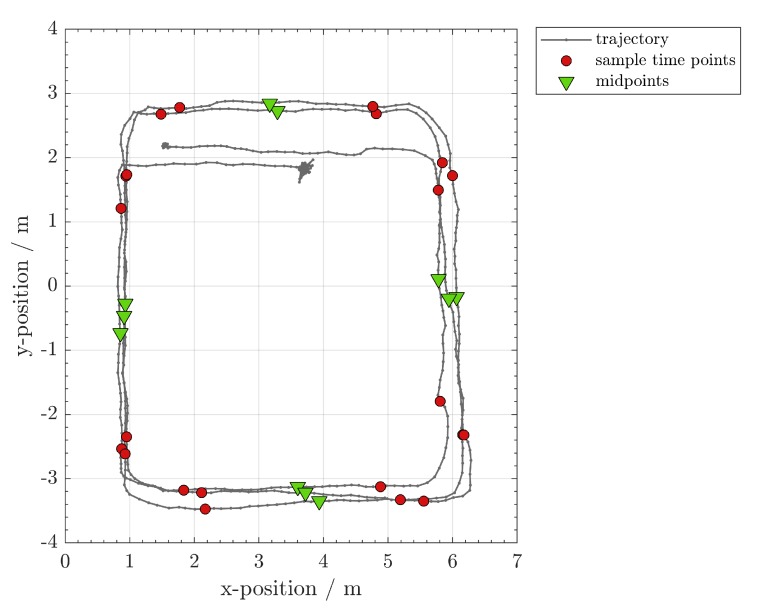
Using the inertial measurement unit, one can detect the straight lines. The shown trajectory is estimated using ultrasound localization and known anchor positions. While this is not the case, it shows how the target moved during the calibration phase. The magenta points are critical points which define the starting and ending points of the straight lines.

An example is given in Figure 7. We can observe several peaks and saddles in the $\omega_{z}$ curve. The peaks occur when the vehicle turns its direction and the saddles indicate that the beacon is moving in a straight direction. The magenta points denote the endpoints of the straight-moving interval, while the green points are the midpoints of the time interval. In Figure 8, we can see how the straight lines are successfully detected in a real trajectory. We name the endpoints of the time interval in which the target moves in a line as the *critical time points*, their location are the *critical waypoints* and the corresponding TDoA measurements the *critical TDoA sequences*. In this work, a *waypoint* refers to a location sample on the trajectory. Each waypoint is associated with a TDoA sequence.

### 4.3. Direction Adjudgment

In order to reduce the computational complexity and the calibration effort, we first assume that we have estimated the distance from the encompass anchor to three pivotal anchors. Then, we have four anchors, which are enough to estimate the position of the target using TDoA. Two of these anchors will have a fixed position and two of them will be located in circles; as it can be seen in Figure 6. In order to find which are the most likely positions of the anchors inside the circles, we use the IMU data. For every possible position of the anchors, we estimate the positions of the target using TDoA. In order to do this, we use a closed-form [26] formulation. From the TDoA estimations, we extract the forward directions and compare them with the rotations measured by the gyroscope. The similarity between the trajectory measured by the IMU and the one estimated with the TDoA measurements lets us know which are the most likely anchor positions.

If a target turns, then moves straight and then turns, the line is defined by the critical points $b_{j}^{h}$,$b_{j}^{e}$ which denote the starting and ending points of the line. Then, we can estimate the direction ${\hat{\theta }}_{j}$ for the straight segment *j* with

(19) $$\begin{array}{c} {\hat{\theta }}_{j}=\arctan\left({\hat{b}}_{j}^{e}-{\hat{b}}_{j}^{h}\right) \end{array}$$

where ${\hat{\theta }}_{j}$ is the estimated direction of the straight segment *j*, ${\hat{b}}_{j}^{h}$ is the estimation of the start critical point and ${\hat{b}}_{j}^{e}$ is the estimation of the end critical point.

#### Rotation Calculation

In order to estimate the rotation, we integrate the horizontal rotation rate in the time interval $\left[t_{m1},t_{m2}\right]$. We formulate it in a generalized time interval $\left[t_{m,j},t_{m,j+1}\right]$ as

(20) $$\begin{array}{c} \Delta \theta_{j}=\int_{t_{m,j}}^{t_{m,j+1}}\omega_{z}\left(t\right)\;dt, \end{array}$$

where $\Delta \theta_{j}$ is the measured rotation between the straight segments *j* and j+1, and $\omega_{z}$ is the rotation rate around *z*-axis of the vehicle frame.

In the end, we apply

(21) $$\begin{array}{c} \left|\theta_{j+1}-\theta_{j}-\Delta \theta_{j}\right|<\theta_{th} \end{array}$$

to judge the direction, where $\theta_{th}$ is a chosen threshold. If the measurements fulfill Equation (21), we consider the guessed anchor positions to be feasible.

**Figure 9:**
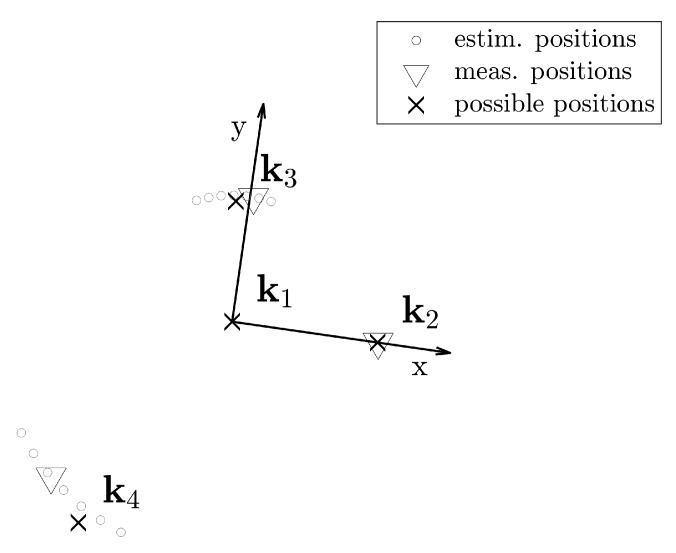
Possible space and initialization after direction adjudgment. Notice that the subspace of possible solutions is reduced from circles to arcs and then to points $k_{3}$ and $k_{4}$, which are used for initialization.

The trajectory may consist of several straight segments. Each pair of adjacent straight segments forms a constraint of the form formulated in (21). We sum up the number of satisfied constrains for each of the guessed configurations. In the end, we keep only the guesses with the largest number of satisfied constrains and exclude the others. We provide an example of the reduced possible spaces after direction adjustment in Figure 9. Note that the arc-shaped possible space is still not the initialization. We average the remaining $\phi_{k}^{i}$ of an anchor *i* and initialize it as $k^{-}$, where ${\bar{\phi }}_{k}^{i}$ is the mean of the feasible angles. We provide an example of the result in Figure 9. The hollow circles are the remaining anchor positions while the points used for initialization are denoted as filled circles in Figure 9.

In this section, we use the closed-form TDoA solution presented by Bucher and Misra in [26] to estimate the target positions. This closed-form solution requires four anchors. When the number of anchors increases, the possible solutions increase exponentially, which makes the direction adjudgment inefficient. Therefore, we introduce a new method to initialize the additional anchors.

### 4.4. Initialization of Additional Anchors

With the first four anchors initialized, we can use the initialized positions to approximate the true positions of the first four anchors. The subspaces of possible positions for the additional anchors are also circles. First, we randomly select several TDoA sequences and estimate the corresponding sender positions with the approximation of the first four anchors. With the estimated sender positions, we reconstruct the TDoA measurements that each additional anchor would receive and compare them with the measured TDoAs. Repeating this process over all the possible positions of the additional anchors, we choose the possible positions whose resulting forward directions comply best with the gyroscope measurements for initialization.

**Figure 10:**
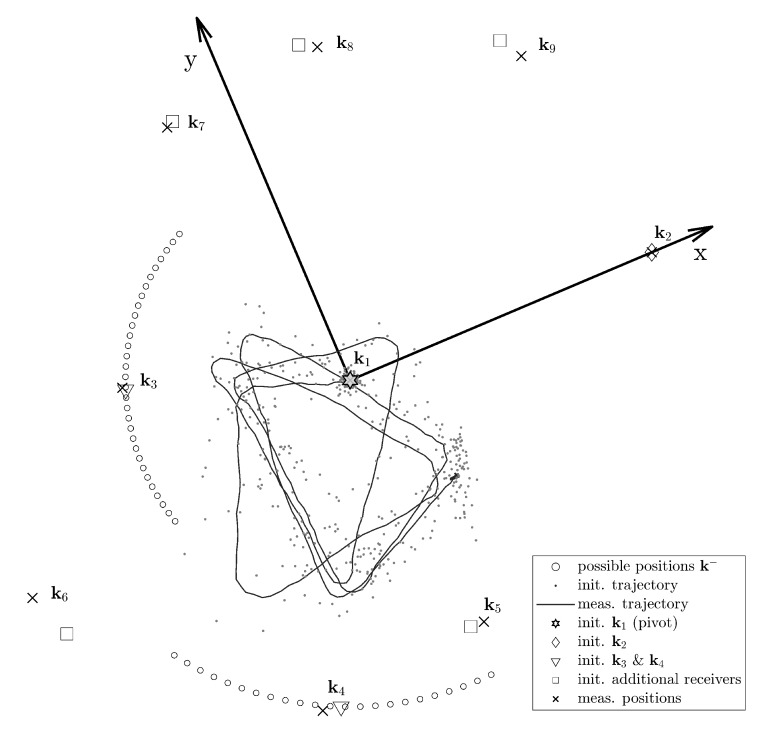
Initialization with direction adjudgement example.

To initialize the trajectory, we randomly choose a configuration from the possible spaces for each of the waypoints on the trajectory and estimate the sender positions with the closed-form solution. Figure 10 gives an example of the initialization results. We choose a central anchor $k_{1}$ as the origin and a randomly selected encompassing anchor $k_{2}$, through which we define the positive *x*-axis. The position estimation of the next anchors $k_{3}$ and $k_{4}$ is selected from the reduced solution sub-space. We estimate the initial positions of these latter two anchors from the average coordinates in the polar system.

### 4.5. Optimization

Once we have an initialization for the position of the anchor nodes and trajectory of the sender we minimize the following quadratic error function:

(22) $$\begin{array}{c} E_{ij}^{\mathrm{TDoA}}=r_{bi}-r_{b1}-c\;\tau_{1i}. \end{array}$$

The objective function is the squared sum of TDoA residuals, which is formulated as

(23) $$\begin{array}{c} \underset{k_{i},b_{j}}{\mathrm{error}}\sum_{i=2}^{N_{k}}\sum_{j=1}^{m}{\left(E_{ij}^{\mathrm{TDoA}}\right)}^{2}. \end{array}$$

In order to search for the minimum value of the objective function, we apply the Levenberg-Marquart algorithm.

## 5. Simulations

We designed and performed a simulation in order to verify that the direction adjudgment can be generalized to different anchor topologies.

The simulation depends on both the IMU and TDoA inputs. However, we are not able to simulate the IMU measurements since the theoretical models often differ highly from reality. Therefore, we use real IMU measurements. Based on the randomly generated anchor positions and the recorded beacon trajectories, we estimate the theoretical values of TDoA measurements and impose Gaussian distributed errors on the theoretical values. The assumption of a Gaussian distribution does not hold, as was also shown by Cui et. al. [27], but for this work we estimated the resulting error as small enough to be neglected at this stage.

### 5.1. System Error Distribution

**Figure 11:**
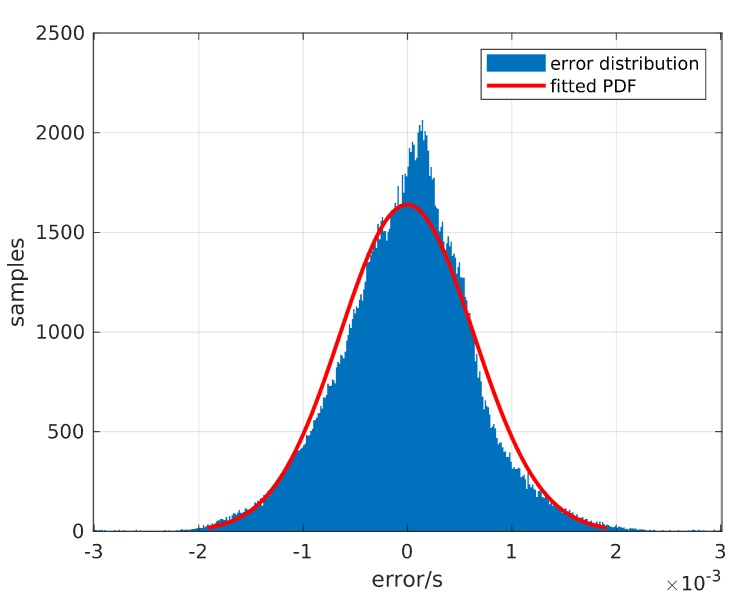
Fitting the TDoA error with a Gaussian distribution.

First, we have to determine the error level of the TDOA localization system before starting the simulation. Therefore, we estimate the theoretically expected TDoA values using the real trajectories recorded by a Motion Capture system, which has submillimeter accuracy and precision, and the true anchor positions. We compared the TDoA measurements and the expected values in 200,138 TDoA measurements to estimate the measurement errors (see Figure 11). Fitting the error distribution into the Gaussian distribution, we obtained a Gaussian distribution with $\mu_{\mathrm{TDoA}}=-0.006\;ms$ and $\sigma_{\mathrm{TDoA}}=0.637\;ms$. Since μ≪σ, we regard the offset of the error distribution to be 0.

Actually, the localization system measures the timestamp of the signal of arrival. The TDoA is the subtraction of two timestamps measured by the different anchors. Assuming that the errors of the timestamp measurements from all the anchors comply with the same Gaussian distribution, i.e., $e_{t}∼N\left(\mu_{t},\sigma_{t}^{2}\right)$, we have:

(24) $$\begin{array}{c} e_{\mathrm{TDoA}}=e_{t,i}-e_{t,1}∼N\left(\mu_{t},\sigma_{t}^{2}\right)+N\left(-\mu_{t},\sigma_{t}^{2}\right)=N\left(0,2\sigma_{t}^{2}\right). \end{array}$$

Therefore, the standard deviation is calculated as:

(25) $$\begin{array}{cc} \sigma_{t} & =\sqrt{\frac{1}{2}\;\sigma_{\mathrm{TDoA}}^{2}}=0.45\;ms. \end{array}$$

### 5.2. Performance Comparison of DA vs. RI

**Figure 12:**
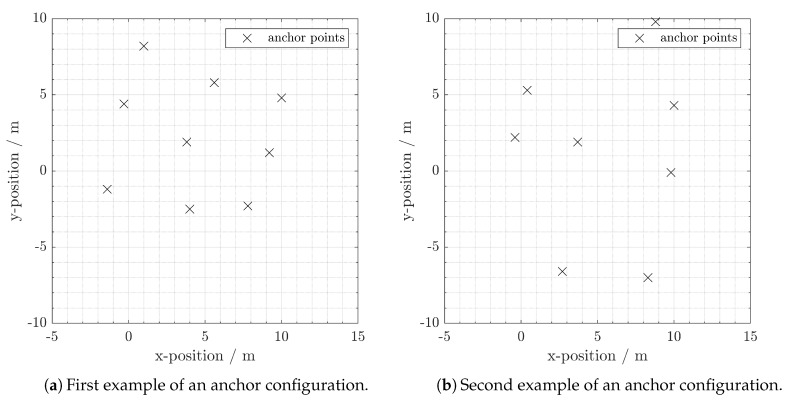
Two examples of the 2000 simulated anchor configurations.

**Figure 13:**
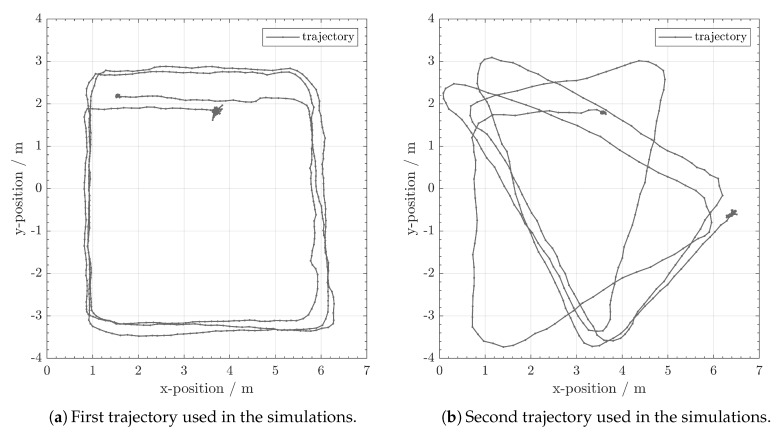
The two trajectories shown in (**a**) and (**b**) respectively are used in the simulations to emulate a moving beacon.

We randomly generated 1000 different anchor configurations. Each configuration contains nine anchors. In reality, one would not install two anchors very close to each other. Therefore, we set the minimum distance between two arbitrary anchors to be three meters. Figure 12 gives examples of simulated anchor configurations. In the simulation, we use two target trajectories (see Figure 13). Since we generate 1000 anchor configurations, we will have 2000 different simulation scenarios. In the end, we compare the performance between DA-SC and RI-SC over all the scenarios.

One of the performance indicators is the success rate. In the self-calibration problem, the anchor positioning error larger than 0.5 m is mainly due to local minima. Therefore, we have the following definition of success:

**Definition** **1**. *Success of Self-calibration: A self-calibration attempt succeeds if the following condition holds*

(26) $$\begin{array}{c} \max\;\left(\{∥{\hat{k}}_{i}-k_{i}∥\}\right)<0.5\;m \end{array}$$

*where ${\hat{k}}_{i}$ is the estimation of the anchor position and $k_{i}$ denotes the true position.*

The direct self-calibration results are likely to be in a different coordinate system to the true anchor positions. The estimations are rotated and translated according to the reference system before the comparison.

Since direction adjudgment is a deterministic process, we evaluate only its single success rate from the first attempt. Table 1 gives the success rate at the first attempt between direction adjudgment (DA-SC) and random initialization (RI-SC) for the two different trajectories.

**Figure 14:**
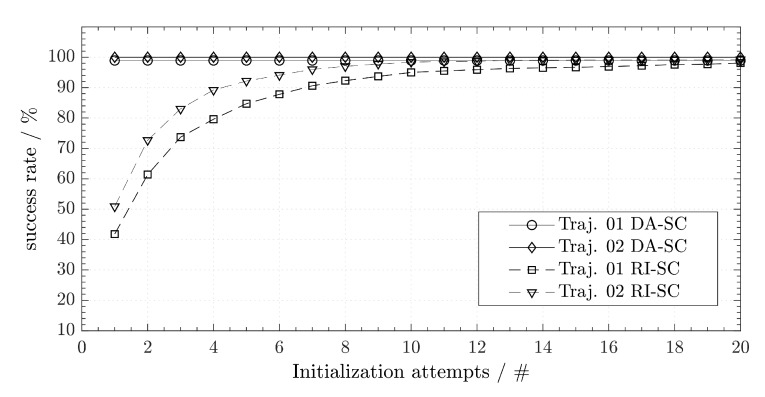
Comparison between the success rate of different initialization methods.

In addition, Figure 14 shows the difference of performance between DA-SC and RI-SC. The corresponding success rates given in Figure 14 and Table 1 are the percentage of simulated topologies that were successfully estimated by DA-SC. In contrast, randomized initialization is a stochastic process. We repeat the RI-SC for 20 times redoing the unsuccessful cases and count the accumulative success rate after each repetition. We observe that the success rates of DA-SC are nearly 100%. Comparatively, the success rates of RI-SC can only reach this level after 10–20 repetitions.

**Figure 15:**
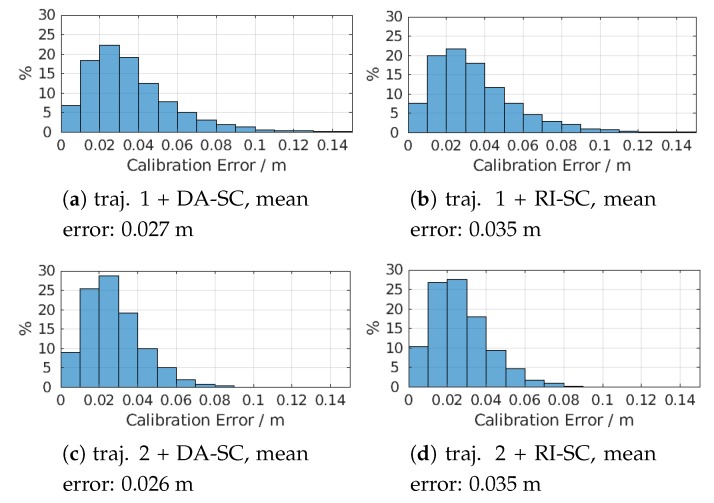
Error distributions for different simulation configurations.

As shown in Figure 15, since we use the same objective function, the calibration results have a similar error and the resulting mean errors have nearly the same value.

## 6. Experiments of IMU-Assisted Self-Calibration

We performed experiments for the verification and further analysis of the simulation results.

### 6.1. Locales and Set-Up

We present measurements from the two seperate locales *Hangar* (see Section 6.1.1) and *Messe* (see Section 6.1.2). These installations have different characteristics and, therefore, allow us to test our proposed algorithm under different conditions. The receivers are placed at a larger height in the Messe, which increases the dilution of precision. However, in the experiment in Hangar there are more obstacles and, therefore, the signals can be reflected or occluded.

#### 6.1.1. Experiments in the Hangar

**Figure 16:**
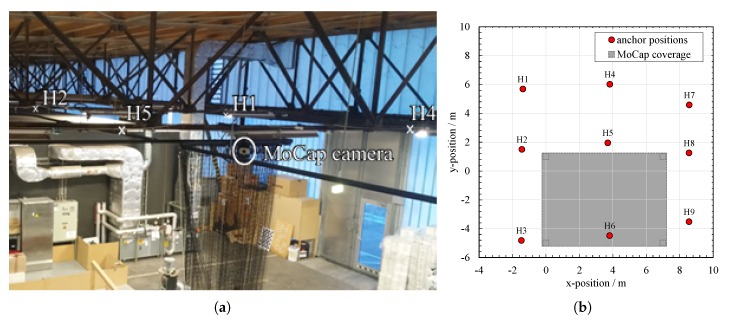
The positions of the anchors H1-9 in the Hangar experiments with an exemplary trajectory of experiment 09. There are 12 anchors in hangar of which we use only 9. The shaded area indicates the coverage of the MoCap system. (**a**) Photo of the Hangar ceiling installation. (**b**) Anchor positions and MoCap coverage.

The anchors in the Hangar are mounted in a height of appoximately $z_{H}\approx 4.9\;m$ above the concrete floor to steel beam girders and powered permanently by wire. We measured the real positions of the anchors with a Total Station *Topcon GPT-8203A* for reference. A part of the anchor set-up is shown in Figure 16a for illustration. We use the Motion Capture (MoCap) system *MotionAnalysis Cortex* with *Raptor-E* cameras to record the true trajectory with sub-millimeter precision. The MoCap system only provides reliable positions in the area between the cameras, which results in a limited coverage area that is considerably smaller than the one of ASSIST. Figure 16b is the top-view of the Hangar experimental area with anchor positions, where the grey area indicates the MoCap coverage.

#### 6.1.2. Experiments in the Exhibition Hall

**Figure 17:**
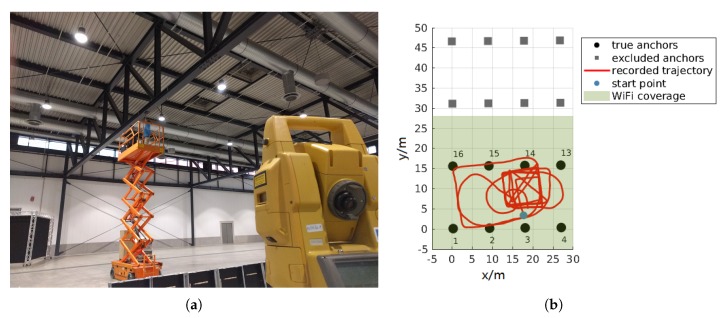
The anchor positions in the exhibition hall 3 of Messe Freiburg with an exemplary trajectory of Exp. 16. Notice the error of about 4m in the start point location: The true start point of the trajectory should be closer to anchor M03 as the beacon was placed directly below this anchor. (**a**) Photo of the anchor node installation at the Messe. (**b**) Anchor positions and Exp. 16 trajectory.

We installed 16 anchors in the commercial exhibition hall “Halle 3” of Messe Freiburg at the bottom of the steel girders at a height of z≈7.3 m, as shown in Figure 17a. The smartphone requires Wi-Fi connectivity to synchronize the IMU and ASSIST. Due to the limited Wi-Fi coverage, we can only calibrate eight of the anchors. Figure 17b visualizes the anchor positions and an exemplary recorded trajectory from our experiment.

### 6.2. Experimental Vehicle

**Figure 18:**
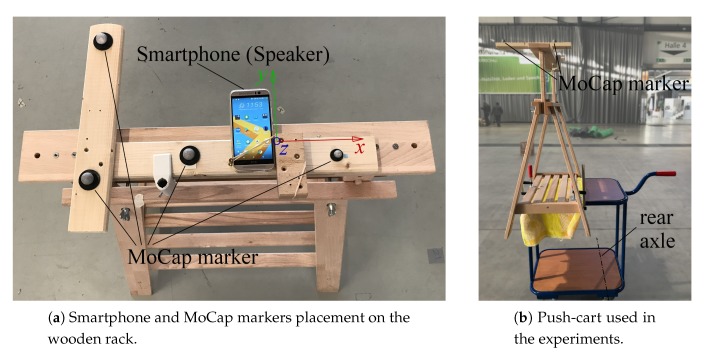
The smartphone on the wooden rack is laterally aligned to the midpoint of the rear axle, with the *x*-axis of the smartphone in parallel to the rear axle.

We use a *HTC One* smartphone for both ASSIST and IMU tracking. The smartphone is fixed to a wooden support rack along with the motion capturing (MoCap) markers, and this is shown in Figure 18a. We align the smartphone to the midpoint between the rear wheels with the *y*-axis of IMU perpendicular to the rear axle. Moreover, we fix it horizontally with its screen facing upwards. As a result, the speaker, which is located above the top of the device’s screen, is suspended in air. This is done to roughly mimic the smartphone being held in front of your chest while moving and to reduce offset errors. This set-up is then fixed to a push-cart as shown in Figure 18b, which we slowly moved through the experimental areas. The cart has two freely rotating front wheels and two fixed-heading rear wheels, known as an Ackermann steering geometry, which is common for wheeled vehicles [28]. All the wheels are nonholonomic which prevent the side slipping. The heading direction of the vehicle’s body is always perpendicular to the rear axle [28], i.e., the moving direction is perpendicular to the rear axle.

### 6.3. Experimental Results

In the anchor self-calibration experiments in the Hangar we calibrate nine anchors. We also validate the direction adjudgment with 17 real-world experiments. Table 2 summarizes the 17 experiments, among which Exp. 01–12 were conducted in Hangar and Exp. 13–17 were in Messe Freiburg. We applied both direction adjudgment self-calibration (DA-SC) and randomized initialization self-calibration (RI-SC) over all experiments. We repeated the RI-SC for 100 times in each experiment, but execute the DA-SC for only once. Table 2 gives the comparison between DA-SC and RI-SC. We list the results of both initialization and optimization for DA-SC. To evaluate the success rate of RI-SC, we repeat the RI-SC for 100 times and calculate the percentage of success attempts. Note that we use Definition 1 again in this section to define the success of self-calibration. The result shows how the success rate is increased while the mean error is similar in both cases, as expected from the simulated environment. Of special interest are the experiments in the Hangar, as their success rate is limited using randomized initialization due to the large height of the receivers, which increases the dilution of precision. It is worth mentioning that the mean error in these cases is calculated with a reduced number of valid attempts. This is the reason why one observes a larger discrepancy in the mean error of both initialization strategies.

## 7. Experiments of Zone Detection with Machine Learning

The aim of this experiment is to compare the performance of each algorithm in whether it correctly associates the presence of beacons with the correct zone.

### 7.1. Experimental Setup

**Figure 19:**
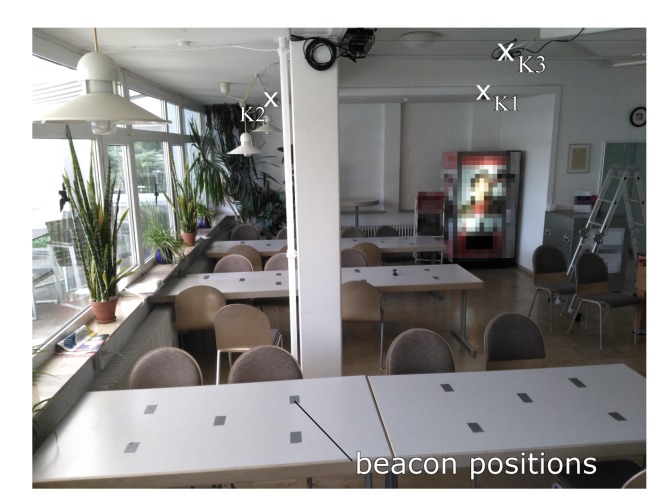
Experimental scenario for the machine learning experiments. A beacon is put on a table approximately at the marked cluster positions and the aim is to correctly estimate the cluster it is in by training each machine learning algorithm with the TDoA measurements of multiple positions on the tables.

In order to test the capability of an artificial neural network to locate the target with unknown anchor positions and a realistic environment, we perform a real experiment in a cafeteria (see Figure 19). We mount seven anchors on the ceiling. Then, we place a sender in 29 different zones on tables, that are marked by tape. The received timestamps at each zone and the zone number serve as the training set for the machine learning algorithms.

### 7.2. Test Set Creation

We create a test set by placing the sender near each of the trained points in order to estimate the percentage of correct associations. We move the sender near every trained point so that we can simulate a real environment, where the sender is not placed exactly in the trained points.

### 7.3. Experimental Results

**Figure 20:**
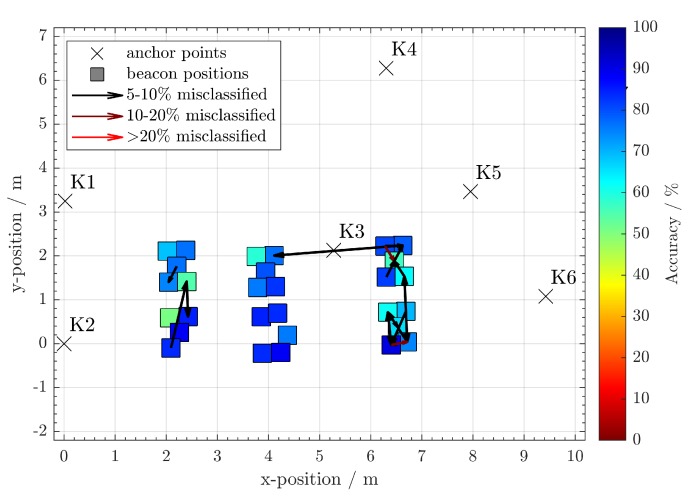
Approximate positions of the clustered beacon positions and the most clear misclassifications using a **neural network**. The colors of the beacon clusters indicate their accuracy of correct assignments.

**Figure 21:**
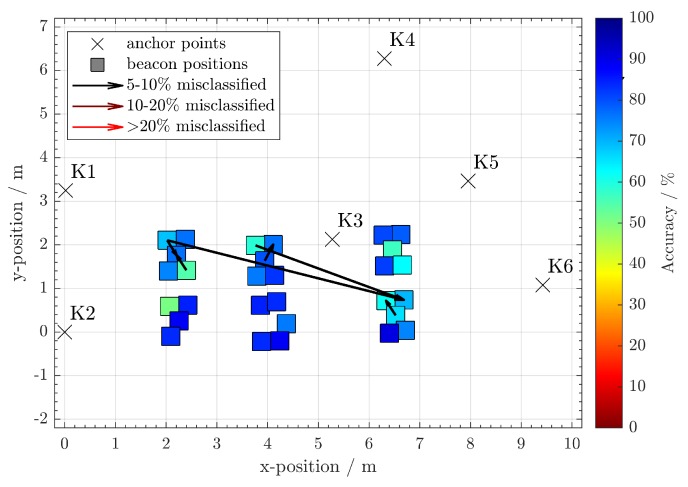
Approximate positions of the clustered beacon positions and the most obvious misclassifications using **random forest**. The colors of the beacon clusters indicate their percentage of correct assignments.

After evaluating the data, there were 91% correct associations using random forest and 88% using a neural network. Note that most of the cluster errors occur in close geometric proximity, and consequently similar TDoA. This is indicated by the arrows in Figure 20 and Figure 21. If one allows a certain geometrical error in the estimations, the number of correct assignments is higher. For example, if only the correct table is to be identified, but not the individual position at the table, only the larger misclassifications beyond the table zones can be considered erroneous. The effect of increased cluster sizes can be seen in Table 3. The random forest achieves throughout the whole experiment a larger number of correct assignments than the artificial neural network. This difference is more noticeable for the smallest cluster size.

From the results in Table 3 we can conclude that machine learning can be used for localization in situation where one wants to avoid manually measuring the positions of the anchors and requires only to detect whether the speaker is in certain regions.

## 8. Conclusions

In this work, we have presented two methods for acoustic indoor localization which do not require manually measuring the positions of the microphones. We have presented a method for estimating the anchor positions using an inertial measurement unit as additional information. This method provides an initial estimation for non-linear optimization algorithms which often fall into local minima. By doing this, we increase the single-shoot success rate. We have shown the performance of our method in both simulations and real-world experiments. The other approach presented in this work consists of using machine learning to identify in which region a speaker is lying. We have shown experimentally how using random forest classification, one can know where the sender is lying with 94 of accuracy defining regions of around 0.8 m${}^{2}$.

However, various open lines for future work remain. When estimating the position of the microphones, we have multiple restrictions on the trajectory for initialization, as one has to start directly beyond one of the anchor nodes and the trajectory must contain straight line segments. In the future, it would be desirable to overcome those restrictions and allow for more general trajectories.

So far, we have assumed to have all the anchors at a known height and performed two-dimensional localization only. An obvious line for future work is, thus, to extend the methods to three dimensions.

Furthermore, the machine learning approach could be extended by training the algorithm with other data such as the amplitude of the received signals or all the reception times of the reflections.

## Figures and Tables

**Table 1:** Comparison between success rates at the first attempt of the two self-calibration methods Direction Adjudgment (DA-SC) and Random Initialization (RI-SC).

	Success Rate/%	
Trajectory	DA-SC	RI-SC
1	98.9	41.8
2	100	50.9

**Table 2:** Performance comparison between DA-SC and RI-SC in real experiments. The success rates of RI-SC is the percentage of success attempts over 100 repetitions.

	DA-SC	RI-SC	
Experiment	Mean Error/m	Success Rate	Mean Error/m
01	0.12	57	0.13
02	0.12	80	0.11
03	-	*error*	-
04	0.13	17	0.14
05	0.10	25	0.11
06	0.06	90	0.07
07	0.15	23	0.15
08	0.09	85	0.09
09	0.13	67	0.11
10	0.13	84	0.10
11	0.08	69	0.09
12	0.12	67	0.11
Overall	0.11	60	0.11
13	0.28	5	0.30
14	0.10	1	0.36
15	0.20	0	-
16	0.10	0	-
17	0.19	0	-
Overall	0.17	1	0.33

**Table 3:** Correct assignments using an artificial neural network and a random forest.

Cluster	Approx. Dimensions	Neural Network	Random Forest
I	0.03 m${}^{2}$	87.8%	90.5%
II	0.80 m${}^{2}$	94.1%	94.3%
III	7.15 m${}^{2}$	95.5%	96.8%
